# Effects of Stimulus-Driven and Goal-Directed Attention on Prepulse Inhibition of Brain Oscillations

**DOI:** 10.3389/fnhum.2016.00390

**Published:** 2016-07-29

**Authors:** Agnès Annic, Jean-Louis Bourriez, Arnaud Delval, Perrine Bocquillon, Claire Trubert, Philippe Derambure, Kathy Dujardin

**Affiliations:** ^1^University of Lille, INSERM U1171 - Degenerative and Vascular Cognitive DisordersLille, France; ^2^Department of Clinical Neurophysiology, Lille University Medical CenterLille, France; ^3^Department of Neurology and Movement Disorders, Lille University Medical CenterLille, France

**Keywords:** prepulse inhibition, brain oscillations, continuous performance test, attention, time-frequency analysis

## Abstract

**Objective**: Prepulse inhibition (PPI) is an operational measure of sensory gating. PPI of cortical response to a startling pulse is known to be modulated by attention. With a time-frequency analysis, we sought to determine whether goal-directed and stimulus-driven attention differentially modulate inhibition of cortical oscillations elicited by a startling pulse.

**Methods**: An electroencephalogram (EEG) was recorded in 26 healthy controls performing an active acoustic PPI paradigm. Startling stimuli were presented alone or either 400 or 1000 ms after one of three types of visual prepulse: to-be-attended (goal-directed attention), unexpected (stimulus-driven attention) or to-be-ignored (non-focused attention). We calculated the percentage PPI for the auditory event-related spectral perturbation (ERSP) of theta (4–7 Hz), alpha (8–12 Hz), beta1 (13–20 Hz) and beta2 (20–30 Hz) oscillations and changes in inter-trial coherence (ITC), a measure of phase synchronization of electroencephalographic activity.

**Results**: At 400 ms: (i) PPI of the ERSP of alpha, theta and beta1 oscillation was greater after an unexpected and a to-be-attended prepulse than after a to-be-ignored prepulse; and (ii) PPI of beta2 oscillations was greater after a to-be-attended than a to-be-ignored prepulse. At 1000 ms: (i) PPI of alpha oscillations was greater after an unexpected and a to-be-attended prepulse than after a to-be-ignored prepulse; and (ii) PPI of beta1 oscillations was greater after a to-be-attended than a to-be-ignored prepulse. The ITC values did not vary according to the type of prepulse.

**Conclusions**: In an active PPI paradigm, stimulus-driven and goal-directed attention each have differential effects on the modulation of cortical oscillations.

## Introduction

### Overall Background

Filtering out irrelevant information is a crucial way of protecting the cognitive resources required for goal-directed activities. One of the physiological indices of these protective neural processes is referred to as prepulse inhibition (PPI, an index of sensorimotor gating). It corresponds to the attenuation of the amplitude of a startle reflex to an intense stimulus (called the pulse) when a weaker, non-startling stimulus (the prepulse) precedes the pulse by approximately 30–500 ms. The prepulse attenuates not only motor responses (e.g., the eye-blink reflex) but also cortical responses to a sound pulse, such as the N100 and P200 components of the auditory evoked potential (AEP; Perlstein et al., 1993, 2001), or evoked-brain oscillations (Kedzior et al., 2006, 2007), which might be valuable for understanding the impairment of the mechanisms of sensory gating in the context of disorders such as schizophrenia (Inui et al., 2012).

### PPI and Attention

PPI is mediated by a broad network that includes cortical regions known to be involved in attention, namely fronto-striato-thalamic circuitry, cingulate cortex, inferior parietal lobe/supramarginal gyrus and superior temporal lobe (Swerdlow et al., 2001; Kumari et al., 2003, 2005; Campbell et al., 2007; Hazlett et al., 2008a). Moreover, attention can modulate the magnitude of PPI. Indeed, several researchers have reported that in an active PPI paradigm (when participants are explicitly asked to attend to the prepulse), PPI is greater after a to-be-attended than after a to-be-ignored prepulse (Dawson et al., 1993; Filion et al., 1993). However, attention can be either goal-directed (i.e., focused on relevant signals derived from task demands) or stimulus-driven (i.e., captured by salient properties of stimuli that are sometimes irrelevant for the task; Desimone and Duncan, 1995; Kastner and Ungerleider, 2000). In this respect, our recent study of the N100 and P200 components of the AEP demonstrated that stimulus-driven attention and goal-directed attention each have specific effects on PPI (Annic et al., 2014). Namely, PPI of the N100 component was specifically enhanced by stimulus-driven attention while goal-directed attention magnified PPI of the P200 component.

### Time-Frequency Analysis of EEG

Some approaches consider that brain activity consists of neuronal ensembles oscillating within particular frequency bands. Hence, electroencephalogram (EEG) activity can be described in a “time-frequency domain” using two measures: event-related spectral perturbation (ERSP) and inter-trial coherence (ITC; Makeig et al., 2004). The ERSP corresponds to event-related changes in the power spectrum over time, and thus reflects the number of neurons that discharge synchronously. The ITC is a measure of phase synchronization of the EEG activity from one trial to another at particular time intervals and frequencies. It ranges from 0 (absence of synchronization) to 1 (perfect synchronization) and reflects the extent to which a stimulus causes changes in phase synchrony or induces phase re-setting (Delorme and Makeig, 2004). Evoked activities are time-locked and phase-locked with respect to the stimulus onset; in contrast, induced activities are time- but not phase-locked (Pfurtscheller and Lopes da Silva, 1999). In other words, at a given time and frequency, the presence of both ERSP and ITC close to 1 is indicative of evoked activity, whereas ERSP activity with an ITC close to 0 reflects an induced activity.

The time-frequency analysis of EEG activity may thus contribute to a better understanding of the neuronal oscillations that underlie information processing in the brain. Indeed, cortical oscillations have been linked to various cognitive functions: theta oscillations (4–7 Hz) have been linked to focused attention (Basar et al., 2001), memory performance (Klimesch, 1999), learning (Caplan and Glaholt, 2007), cognitive control and working memory (Hanslmayr et al., 2008); alpha oscillations (8–12 Hz) were reportedly involved in attentional and memory processes (Klimesch, 1999) and are considered to have an active role in inhibitory control (Klimesch et al., 2007); beta oscillations (13–30 Hz) were shown to be involved in goal-directed attention processes (Buschman and Miller, 2007), and in somatosensory and higher-level neural information processing (Bibbig et al., 2001; Cheron et al., 2007; Hong et al., 2008); lastly, lower beta band oscillations (13–20 Hz, referred to as beta1) were also associated with the response to novel stimuli (Haenschel et al., 2000).

### Objectives

Given these links between cortical oscillations and cognitive processing, the study of PPI on this type of cortical marker could be an interesting way of investigating sensory gating. Kedzior et al. (2006, 2007) used a passive PPI paradigm to demonstrate PPI of auditory-evoked theta, alpha and gamma oscillations. To the best of our knowledge, no-one has investigated sensory gating and its attentional modulation with respect to spectral power and phase consistency. The main objectives of the present study using an active paradigm (similar to that reported by Annic et al., 2014) were to examine: (i) whether a prepulse can attenuate the theta, alpha and beta oscillations elicited by a pulse; and (ii) whether goal-directed attention and stimulus-driven attention to this prepulse differ in their modulation of PPI. To this end, we used time-frequency analysis (measuring ERSP and ITC) to study both changes in spectral power and phase-locking. We hypothesized that the degree of inhibition of spectral power across all frequency domains would depend on the prepulse type, with: (i) greater inhibition after a prepulse on which attention was focused (a to-be-attended prepulse or an unexpected prepulse) than after a to-be-ignored prepulse; and (ii) greater inhibition after a to-be-attended prepulse than after an unexpected prepulse. We also verified that inhibition of spectral power was not due to modification in phase synchronization.

## Materials and Methods

### Participants

The study population was the same as in our previous study (Annic et al., 2014); it comprised 26 right-handed, healthy volunteers (10 females, 16 males; mean (standard deviation, SD) age: 22.4 (*2.7*) years). All underwent a semi-structured interview and those with a history of neurological or psychiatric disorders were excluded, as well as those taking psycho-active drugs, including tobacco or cannabis. Subjects with a history of visual or auditory impairments were excluded from the study. All participants gave their written informed consent to participation. The study protocol was approved by the local institutional review board (“Comité de Protection des Personnes Nord-Ouest IV”, Lille, France; reference: 2008-006842-25).

### Task

**Figure 1:**
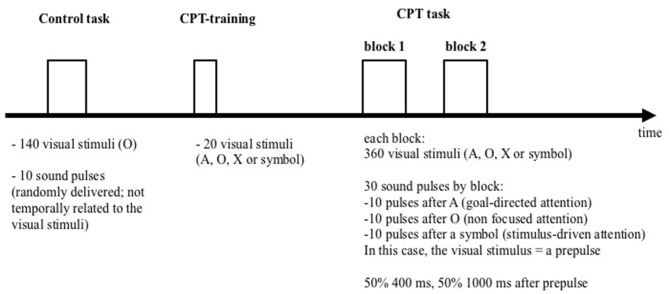
**Representation of the experimental procedure**.

Subjects were comfortably seated and watched a 17″ computer monitor placed 150 cm in front of them. Figure 1 schematically summarizes the procedure which was fully described previously (Annic et al., 2014). Each session included a control task and a startle-continuous performance test (CPT). The startling acoustic stimulus (the pulse) was a 110 dB, 40 ms burst of white noise with a near instantaneous rise/fall time. It was presented binaurally through headphones (TDH39), during the control task and the startle-CPT. Prior to the beginning of each task, the subjects were told that they would occasionally hear a brief burst of noise (the pulse) through the headphones but did not need to pay attention to it.

During the control task, participants were instructed to watch a series of 140 “O” letters (blue letter on a gray screen; 1.57″ high and 1.37″ wide) presented during 29 ms, with a mean 1400 ms inter-stimulus interval (range: 1300–1500 ms). Ten pulses were randomly delivered during the presentation, with an inter-pulse interval ranging from 18 to 30 s (mean: 24 s). The pulses were not temporally related to the visual stimuli.

**Figure 2:**
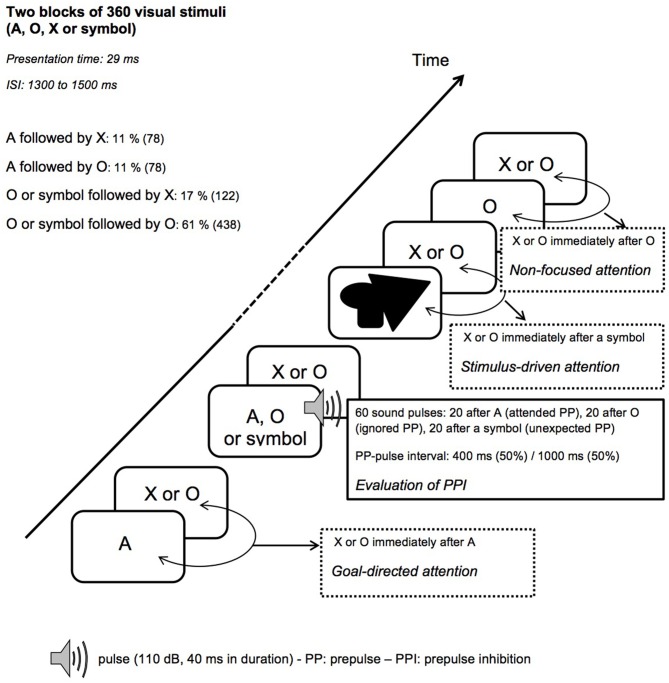
**Schematic representation of the startle-continuous performance test (CPT) task.** ISI, interstimulus interval.

After the control task, participants performed two blocks of 360 trials of the startle-CPT task (Figure 2; for a detailed description, see Annic et al., 2014). During this task, “O”, “A”, “X” letters and meaningless symbol were presented (with the same presentation time and inter-stimulus interval as in the control task). Participants were instructed to press a response button as quickly as possible with the right index finger every time they saw the letter “X” immediately after the cue letter “A”. Participants were not informed that the symbols might appear among the letters. During each block of the CPT task, 30 auditory pulses were delivered: 10 after the letter “A” (the to-be-attended prepulse), 10 after the letter “O” (the to-be-ignored prepulse) and 10 after a symbol (the unexpected prepulse, involuntarily capturing the participant’s attention). Fifteen of these 30 sounds were delivered 400 ms after the visual stimulus (i.e., with a short-lead interval) and 15, 1000 ms after the visual stimulus (i.e., with a long-lead interval). To limit anticipation and habituation, pulses were delivered at variable intervals and with at least 14 s between two pulses. The startle-CPT task blocks were preceded by a practice CPT-only block during which 20 visual stimuli (the letters “O”, “X” and “A”) were presented to the participants, along with one example of an A-X sequence.

The mean response time (in ms), the number of hits and the number of false alarms were recorded.

### Electroencephalographic Recording

An EEG was recorded continuously from 128 scalp locations, using a DC amplifier (ANT Software BV, Enschede, Netherlands) and a Quick-cap^®^ 128 AgCl electrode cap (ANT Software BV) placed according to the 10/05 international system (Oostenveld and Praamstra, 2001) with a linked mastoid reference. A vertical electro-oculogram (EOG) was recorded using two electrodes placed 1.5 cm above and below the axis of the right pupil, in order to detect artifacts related to eye movements. We used Advanced Source Analysis^®^ (ASA) software (ANT Software BV, Enschede, Netherlands) for data acquisition. The EEG and EOG signals were digitized at a sampling rate of 1024 Hz. Electrode impedances were kept below 5 KΩ.

### EEG Analysis

The EEG data were analyzed with ASA^®^ software. The EEG signal was band-pass filtered between 0.1 and 30 Hz, and ocular artifacts were detected and removed off-line using the software’s principal component analysis procedure.

#### ERSP

ERSP was measured using the EEGLab toolbox for MATLAB software (Delorme and Makeig, 2004). Briefly, EEGLab computes the power spectrum over a sliding latency window for each epoch, normalizes each of the latter against the respective mean baseline spectrum and then averages over the trials. Each trial contains samples from −500 ms before and 1000 ms after the pulse. The 200 ms interval prior to the pulse was used as the baseline for computing the ERSP. The ERSP image provides a color code with each image pixel indicating the power (in dB) achieved at a given frequency *f* and latency *t* relative to the pulse onset. Typically, for *n* trials, if *F*_k_ (*f, t*) is the spectral estimate of trial *k* at frequency *f* and time *t*, then:

$$ERSP\left(f,t\right)\;=\;20\log\frac{\sum_{k\;=\;1}^{n}\left|F_{k}(f,t)\right|}{\sum_{t\in baseline}\sum_{k\;=\;1}^{n}\left|F_{k}(f,t)\right|}$$

*F*_k_ (*f, t*), calculation was based on Morlet wavelet decomposition. Using the settings in EEGLab, cycles were set to [2.5, 0.5], i.e., 2.5 cycles at the lowest frequency (4 Hz) with and 9.375 cycles at the highest frequency (30 Hz). A baseline-normalized ERSP was obtained during the control task and for each lead interval (400 and 1000 ms) after each type of prepulse (“A”, “O” and symbol) during the startle-CPT task. Ten trials per condition were considered.

#### ITC

Following calculation of the ERSP, the ITC was computed in order to: (i) evaluate the effect of the prepulse on intertrial phase variability; and (ii) determine whether any abnormalities in ERSP power were due to impairments in phase variability. To compute ITC, the complex output of the baseline-normalized ERSP was divided by its complex norm (i.e., the absolute value), which was then averaged across trials. The complex norm of this averaged value yields the ITC for different time and frequency points. Using the same notation as above, the ITC is defined as:

$$\text{ITC}\left(f,t\right)\;=\;\frac{1}{n}\sum_{k\;=\;1}^{n}\frac{F_{k}(f,t)}{\left|F_{k}(f,t)\right|},$$

where ∣ · ∣ represents the complex norm.

ITC was obtained during the control task and for each lead interval (400 and 1000 ms) after each type of prepulse (“A”, “O” and symbol) during the startle-CPT task. Ten trials per condition were considered.

#### Evaluation of PPI

Apart from the baseline-normalized spectral power value (expressed in dB), the spectral power was also expressed as an absolute value (mV^2^/Hz). Indeed, to calculate the percentage PPI of the power for a given frequency domain, we chose to use the absolute value of the power in mV^2^/Hz (and not the ERSP value in dB) because the baseline period (used to calculate ERSP) differed between the control session and the startle-CPT session. The percentage PPI of the absolute power for the frequency domains was calculated according to the following equation:

$$\frac{\left(\begin{array}{c} \text{absolute power in the}\\ \text{control session} \end{array}-\begin{array}{c} \text{absolute power in the}\\ \text{test session} \end{array}\right)}{\text{absolute power in the control session}}\times \;100$$

PPI were calculated over the time window 75–300 ms after the pulse (corresponding to the auditory response), for each type of prepulse.

The following frequency domains were assessed: theta (4–7 Hz), alpha (8–12 Hz), beta1 (13–20 Hz), and beta2 (20–30 Hz).

### Statistical Analysis

For time-frequency mapping, we used EEGLab to perform permutation tests with four conditions (control, i.e., pulse alone, “A”, “O” or symbol). The threshold for statistical significance (after Holm’s correction) was set to *p* < 0.05. Thereafter, we analyzed the signal on the location where the amplitude of the response was maximum.

All other analyses were performed with SPSS 16.0 for Windows software (IBM^®^ SPSS^®^, Armonk, New York, NY, USA).

The Kolmogorov–Smirnov test was used to check the normality of data distributions. As sex may influence %PPI, the effect of this variable was checked and further analyses were adjusted consequently.

One-factor repeated-measures analysis of variances (ANOVAs) were performed separately for each lead interval and each frequency domain, with the prepulse (“A”, “O” or symbol) as the within-subject factor for the PPI of ERSP and (“control”, “A”, “O” or symbol) for the ITC. When required, *post hoc* analyses with a Bonferroni correction were performed.

The significance threshold was set to *p* < 0.05 for all analyses.

## Results

All data were normally distributed and are presented as the mean and SD. Mean %PPI values did not significantly differ according to sex (*p* < 0.01, for all analyses). By consequence, further analyses were not adjusted.

### Behavioral Performance

The mean reaction time was 369 (43) ms, the mean percentage of correct answers was 99% (2.5) and the mean number of false alarms was 0.15 (0.37).

### Time-Frequency Analysis

**Figure 3:**
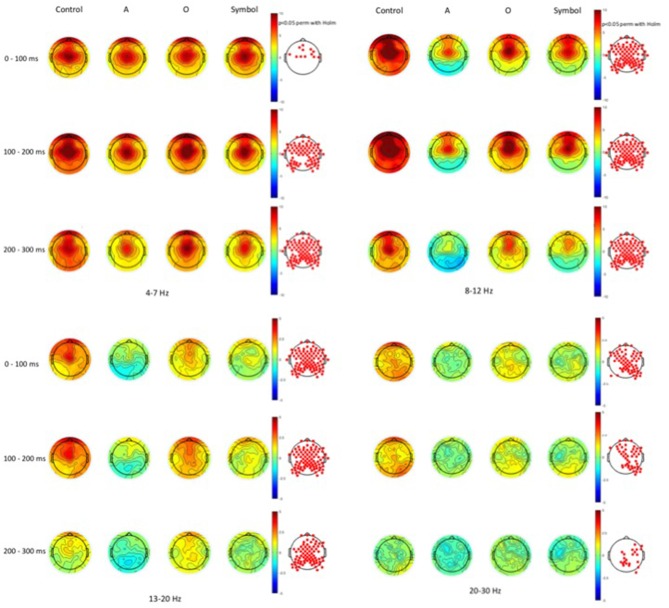
**Scalp topographies of average event-related spectral perturbation (ERSP) values at the 400 ms lead interval in the theta (4–7 Hz), alpha (8–12 Hz), beta1 (13–20 Hz) and beta2 (20–30 Hz) frequency bands.** Decibel changes in spectral power are shown from the pulse presentation (*t* = 0 ms) to 300 ms after, in three 100 ms time epochs. Control: presentation of the pulse alone, A: the pulse is preceded by the “A” prepulse; O: the pulse is preceded by the “O” prepulse; symbol: the pulse is preceded by the symbol prepulse.

**Figure 4:**
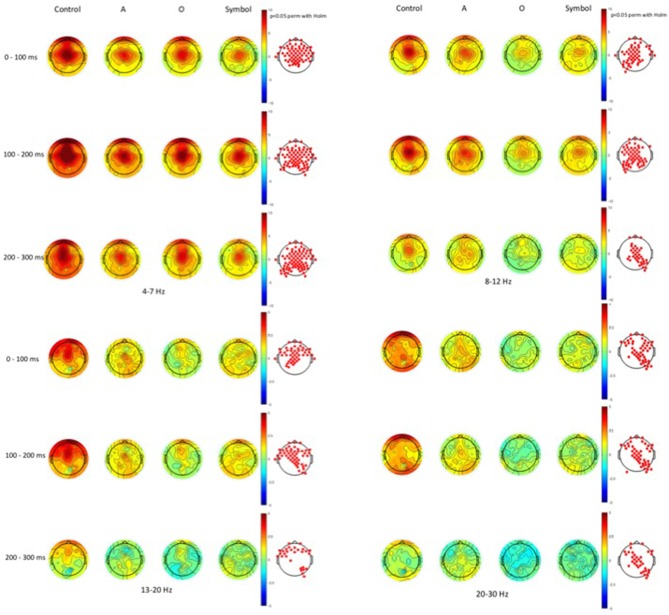
**Scalp topographies of average ERSP values at the 1000 ms lead interval in the theta (4–7 Hz), alpha (8–12 Hz), beta1 (13–20 Hz) and beta2 (20–30 Hz) frequency bands.** Decibel changes in spectral power are shown from the pulse presentation (*t* = 0 ms) to 300 ms after, in three 100 ms time epochs. Control: presentation of the pulse alone, A: the pulse is preceded by the “A” prepulse; O: the pulse is preceded by the “O” prepulse; symbol: the pulse is preceded by the symbol prepulse.

Time-frequency values of the ERSP are displayed as scalp topographies maps during the control session (i.e., when the pulse alone occurs) and during the startle-CPT task [when the pulse is preceded by an “A”, “O” or “symbol” prepulse with a lead interval of 400 ms (Figure 3) and 1000 ms (Figure 4)]. Each map represents the average ERSP values from the pulse onset to 300 ms after (in three 100 ms time epochs).

As displayed on the maps, an occurrence of a pulse was followed by a higher synchronization of cortical rhythms that predominated on the central area and was mainly visible in the theta and alpha frequency bands and to a lesser extent, in the beta1 and beta2 frequency bands. When a prepulse preceded occurrence of the pulse, this synchronization was clearly attenuated but much more when the prepulse was a to-be-attended (“A”) or an unexpected (symbol) stimulus than a to-be-ignored (“O”) stimulus. This phenomenon was observed for both lead intervals, but in an attenuated manner at long-lead interval (1000 ms). Moreover, at short-lead interval (400 ms), when the prepulse was a to-be-attended (“A”) stimulus, a desynchronization was observed 200 ms after the pulse occurrence, mainly in the alpha frequency band and, to a lesser extent, in the beta1 frequency band.

**Figure 5:**
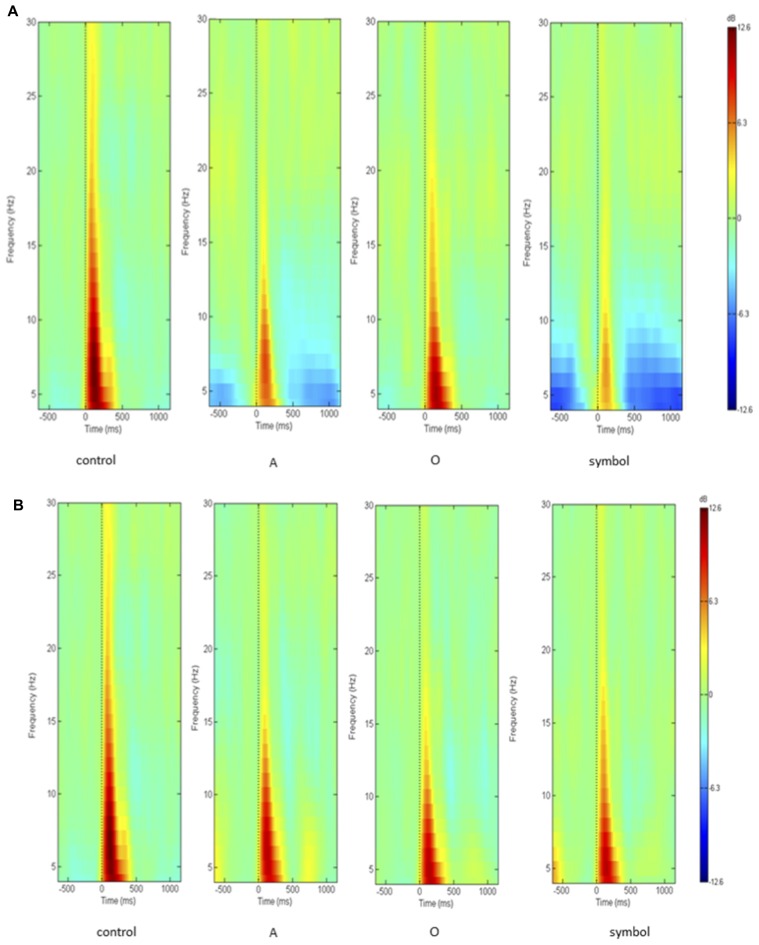
**Grand average of the ERSP over the whole epoch (from 500 ms prior to the pulse presentation to 1000 ms after the pulse, on the *x* axis) and over the whole frequency spectrum (in Hz, on the *y* axis), showing decibel changes in spectral power in response to the pulse presentation (*t* = 0 ms).** Data were recorded at Cz **(A)** with a lead interval of 400 ms; **(B)** with a lead interval of 1000 ms. Control: presentation of the pulse alone; A: the pulse is preceded by the “A” prepulse; O: the pulse is preceded by the “O” prepulse; symbol: the pulse is preceded by the symbol prepulse.

**Figure 6:**
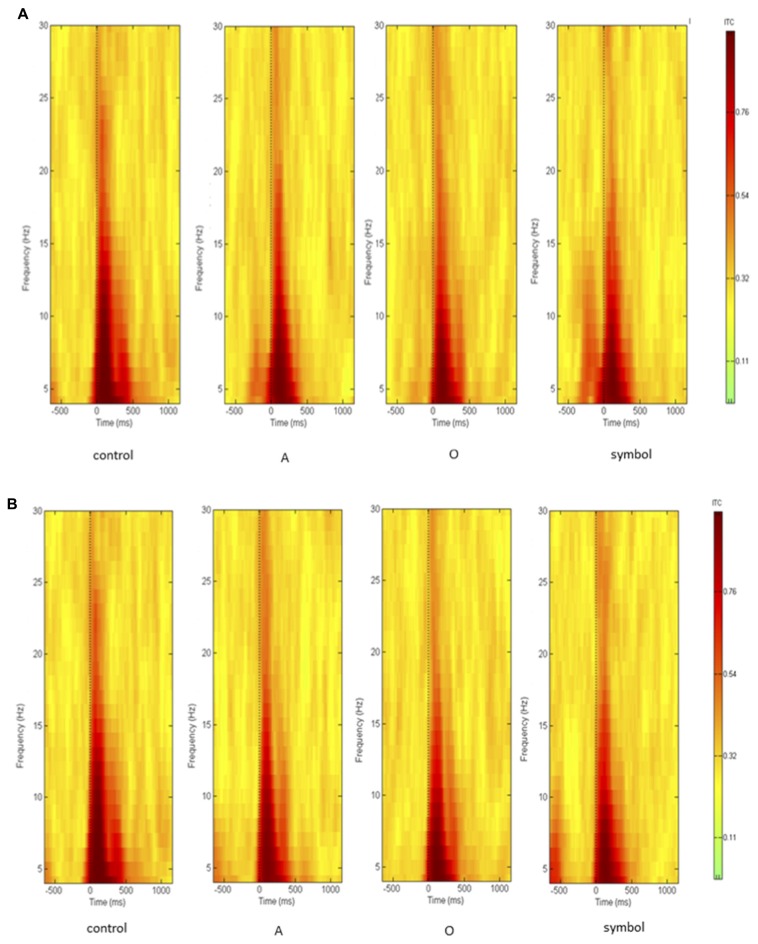
**Grand average of inter-trial coherence (ITC) during the whole epoch (from 500 ms prior to the pulse presentation to 1000 ms after the pulse, on the *x* axis) and over the whole frequency spectrum (in Hz, on the *y* axis), measuring phase consistency across single-trial datasets.** Data were recorded at Cz **(A)** with a lead interval of 400 ms; **(B)** with a lead interval of 1000 ms. Control: presentation of the pulse alone; A: the pulse is preceded by the “A” prepulse; O: the pulse is preceded by the “O” prepulse; symbol: the pulse is preceded by the symbol prepulse.

As activity largely predominated on the central scalp area, further analyses and PPI measures were performed only on the Cz location (where the amplitude of the response to an auditory pulse is known to be maximum (Bruneau et al., 1985)). Figure 5 shows the grand average ERSP obtained at Cz during the control session and during the startle-CPT task with a lead interval of 400 ms (Figure 5A) and 1000 ms (Figure 5B). Similarly, the grand average ITC at Cz is shown in Figure 6 for the same tasks and prepulse-pulse lead intervals.

#### PPI of Spectral Power

**Figure 7:**
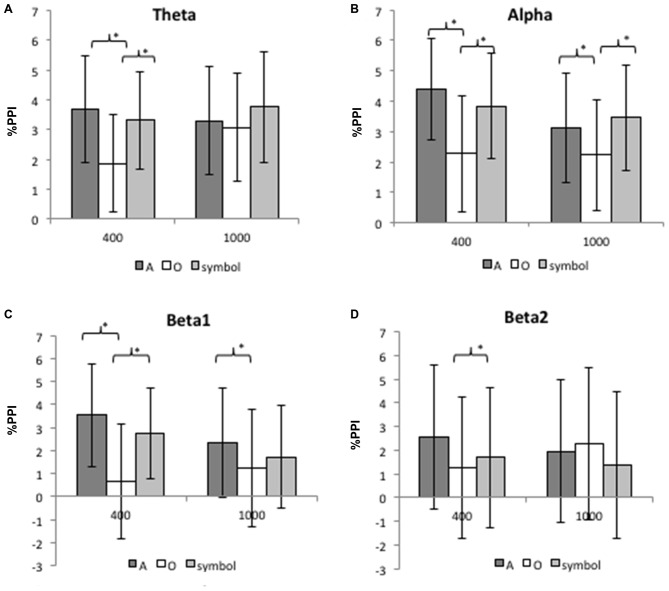
**Mean inhibition of the spectral power for the theta band (A), the alpha band (B), the beta1 band (C) and the beta2 band (D) at 400 and 1000 ms lead intervals for “A”, “O” and symbol prepulses.** Mean inhibition of spectral power was calculated between 75 and 300 ms after pulse presentation. Asterisks indicate significant differences (*p* < 0.05). The bars indicate the standard deviation.

Figure 7 shows the mean (SD) percentage PPI of the spectral power at Cz for each frequency band (theta, alpha, beta1 and beta2) and for lead intervals of 400 ms and 1000 ms.

##### The 400 ms prepulse-pulse interval

For the theta band, an ANOVA revealed a significant main effect of prepulse type (*F*_(2,50)_ = 13.42, *p* < 0.001). Further comparisons (Bonferroni-corrected) revealed that the PPI of the theta rhythm was greater after a prepulse “A” than “O” (*t*_(25)_ = 5.18, *p* < 0.001), and greater after a “symbol” than after a “O” (*t*_(25)_ = 3.84, *p* = 0.001). There was no significant difference between the “A” and “symbol” prepulses (*t*_(25)_ = 0.96, *p* = 0.34).

For the alpha band, an ANOVA revealed a significant main effect of prepulse type (*F*_(2,50)_ = 10.7, *p* < 0.001). Further comparisons (Bonferroni-corrected) revealed that the PPI was greater after a prepulse “A” than “O” (*t*_(25)_ = 5.7, *p* < 0.001) and greater after a “symbol” than “O” (*t*_(25)_ = 2.7, *p* = 0.01). There was no significant difference between the “A” and “symbol” prepulses (*t*_(25)_ = 1.23, *p* = 0.22).

For the beta1 band, an ANOVA revealed a significant main effect of prepulse type (*F*_(2,50)_ = 13, *p* < 0.001). Further comparisons (Bonferroni-corrected) revealed than the PPI was greater after a prepulse “A” than “O” (*t*_(25)_ = 5, *p* < 0.001) and greater after a “symbol” than “O” (*t*_(25)_ = 3.2, *p* = 0.003). There was no significant difference between the “A” and symbol prepulses (*t*_(25)_ = 1.47, *p* = 0.15).

For the beta2 band, an ANOVA revealed a significant main effect of prepulse type (*F*_(2,50)_ = 3.89, *p* = 0.02). Further comparisons (Bonferroni-corrected) revealed that the PPI was greater after a prepulse “A” than “O” (*t*_(25)_ = 2.8, *p* = 0.009). There was no significant difference between the “A” and “symbol” prepulses (*t*_(25)_ = 1.92, *p* = 0.06) nor between the “O” and “symbol” prepulses (*t*_(25)_ = 0.8, *p* = 0.42).

##### The 1000 ms prepulse-pulse interval

For the theta and beta2 bands, there was no significant effect of the type of prepulse.

For the alpha band, an ANOVA revealed a significant main effect of prepulse type (*F*_(2,50)_ = 5.43, *p* = 0.008). Further comparisons (Bonferroni-corrected) revealed that the PPI was greater after a prepulse “A” than “O” (*t*_(25)_ = 2.63, *p* = 0.014), and greater after a “symbol” than “O” (*t*_(25)_ = 3.1, *p* = 0.005). There was no significant difference between the “A” and “symbol” prepulses (*t*_(25)_ = 0.8, *p* = 0.43).

For the beta1 band, an ANOVA revealed a significant main effect of prepulse type (*F*_(2,50)_ = 3.2, *p* = 0.05). Further comparisons (Bonferroni-corrected) revealed that the PPI was greater after a prepulse “A” than “O” (*t*_(25)_ = 2.7, *p* = 0.01). There was no significant difference between the “A” and “symbol” prepulses (*t*_(25)_ = 1.3, *p* = 0.2) nor between the “O” and “symbol” prepulses (*t*_(25)_ = 1.14, *p* = 0.26).

#### The ITC

**Figure 8:**
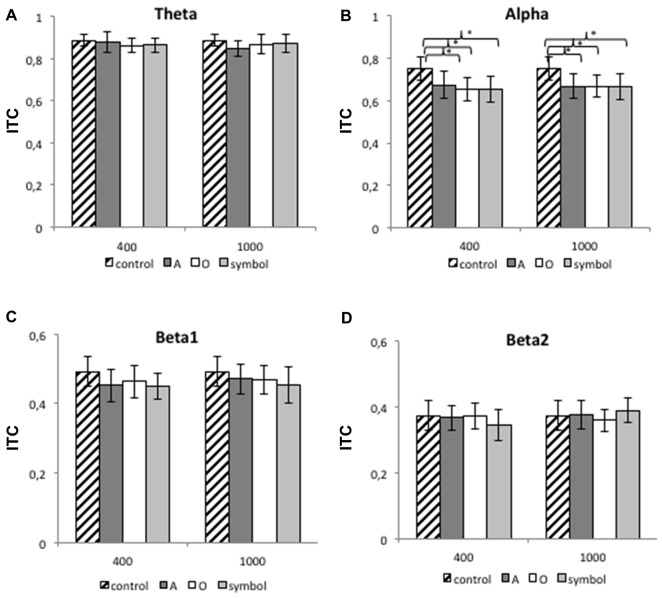
**The mean ITC value for the theta band (A), the alpha band (B), the beta1 band (C) and the beta2 band (D) at 400 and 1000 ms lead intervals for the control condition and for “A”, “O” and symbol prepulses.** The mean ITC value was calculated between 75 and 300 ms after pulse presentation. Asterisks indicate significant differences (*p* < 0.05). The bars indicate the standard deviation.

Figure 8 shows the mean (SD) ITC value at Cz for each frequency band (theta, alpha, beta1 and beta2) and for lead intervals of 400 and 1000 ms.

##### The 400 ms prepulse-pulse interval

For the 400 ms prepulse-pulse interval, there was no significant effect of the condition for the theta, beta1 and beta2 bands, although an ANOVA revealed a significant main effect of condition type for the alpha band (*F*_(3,75)_ = 7.75, *p* < 0.001). Further comparisons revealed than the ITC was greater when the pulse was presented alone (during the control session) than after a “A” (*t*_(25)_ = 3.2, *p* = 0.003), “O” (*t*_(25)_ = 3.8, *p* < 0.001) or “symbol” prepulse (*t*_(25)_ = 3.97, *p* < 0.001). The other differences were not significant (*p* > 0.05).

##### The 1000 ms prepulse-pulse interval

For the 1000 ms prepulse-pulse interval, there was no significant effect of the condition for the theta, beta1 and beta2 bands, although an ANOVA revealed a significant main effect of condition type for the alpha band (*F*_(3,75)_ = 9.3, *p* < 0.001). Further comparisons revealed than the ITC was greater when the pulse was presented alone (during the control session) than after an “A” (*t*_(25)_ = 3.67, *p* = 0.001), “O” (*t*_(25)_ = 4.35, *p* < 0.001) or “symbol” prepulse (*t*_(25)_ = 3.82, *p* < 0.001). The other differences were not significant (*p* > 0.05).

## Discussion

In the present study, we sought to determine the role of attention in the sensory gating process. To this end, we looked at how the EEG oscillations elicited by irrelevant information (the pulse) changed when the said information was preceded by an attention-focusing prepulse.

Our results show that at a prepulse-pulse interval of 400 ms, inhibition of spectral power for the theta, alpha, and beta1 bands was greater after a to-be-attended prepulse and an unexpected prepulse than after a to-be-ignored prepulse. Moreover, inhibition of spectral power in the beta2 band was greater after a to-be-attended prepulse than after a to-be-ignored prepulse, with a trend toward a greater inhibition after a to-be-attended prepulse than after an unexpected prepulse. At a prepulse-pulse interval of 1000 ms, inhibition of spectral power for the alpha band was greater after a to-be-attended prepulse and an unexpected prepulse than after a to-be-ignored prepulse. Inhibition of spectral power for the beta1 was also greater after a to-be-attended prepulse than after a to-be-ignored prepulse. The ITC values did not differ significantly as a function of the type of prepulse.

Hence, by studying brain oscillations, we showed that inhibition of cortical responses produced by an irrelevant stimulus is modulated by attention. Our first starting hypothesis was thus confirmed: inhibition of spectral power in various frequency domains is dependent on the type of prepulse, with a greater inhibition after a prepulse on which attention is focused than after a to-be-ignored prepulse. Regarding our second hypothesis (that inhibition would be greater after a to-be-attended prepulse than after an unexpected prepulse), we only observed a trend towards such an effect for the beta2 frequency band, without significant difference for the other bands. As phase synchrony did not change with respect to the type of prepulse, it can be argued that PPI here is due to a reduction of the number of neurons discharging synchronously.

One of the strengths of our study was its novel design, which enabled us to control for the respective effects of goal-directed attention and stimulus-driven attention.

### Effects at the 400 ms Prepulse-Pulse Interval

At a prepulse-pulse interval of 400 ms, we found that the effects of goal-directed attention and stimulus-driven attention on PPI of the brain oscillations were the same for the theta, alpha and beta1 bands, whereas inhibition of brain oscillations for the beta2 band was greater after a to-be-attended prepulse (involving goal-directed attention) than after an unexpected prepulse (involving stimulus-driven attention). Given that spectral power reflects the number of neurons that discharge synchronously, our results suggest that both stimulus-driven attention and goal-directed attention reduce the number of synchronous discharges in response to an irrelevant stimulus for the theta, alpha and beta1 frequency bands. Moreover, goal-directed attention may reduce the number of neurons that discharge synchronously for the beta2 band. Hence, by using a time-frequency analysis, we confirmed the previous reports (Kedzior et al., 2006, 2007), in which a prepulse inhibited the brain oscillations produced in response to a pulse. However, our present study goes beyond by demonstrating: (i) the role of stimulus-driven and goal-directed attention in inhibition of induced brain oscillations; (ii) the inhibition of beta rhythms (an oscillatory band that has an important role in sensory information processing in general and novelty detection in particular (Haenschel et al., 2000; Kopell et al., 2000; Bibbig et al., 2001; Cheron et al., 2007)) more specifically by goal-directed attention; and (iii) a late desynchronization of the alpha rhythm, specifically induced by goal-directed attention. It is difficult to compare our results with the literature data because our present study is the first to evaluate sensory gating with a PPI paradigm via a time-frequency analysis of neural oscillations. This type of analysis has already been used with a different task (the dual-click paradigm) in healthy controls. In the dual-click paradigm, sounds are presented in pairs. The cortical response to the second sound (S2) is attenuated, relative to the response to the first sound (S1, with a 500 ms inter-stimulus interval). The relative difference in amplitude between S2 and S1 is considered to reflect the suppression (“inhibitory gating”) of redundant and irrelevant stimuli (Braff et al., 1995). The dual-click paradigm has been used to demonstrate that theta, alpha and beta power are lower after S2 than after S1 (Brenner et al., 2009; Smucny et al., 2013). Although our experimental design differed, our results also suggest that sensory gating involves the inhibition of theta, alpha and beta power in response to an irrelevant stimulus. This may indicate that cognitive processing of irrelevant information (which is related to the number of synchronous neuronal discharges) is reduced in this context.

However, in the present study, PPI analyses were focused on the 75–300 ms time window since we wanted to characterize the effect of attention on inhibition of the early cortical oscillations elicited by a startling pulse and our control task showed that the cortical response to this sound pulse was concentrated in this interval (see Figure 5). By consequence, it is likely that we only observed the effect of attention on inhibition of the evoked brain oscillations since ITC is maximal at that time interval. Nevertheless, a careful examination of Figure 5 reveals that beyond 500 ms after the pulse, there was a desynchronization in the theta and alpha bands that only appeared after a to-be-attended or an unexpected prepulse and seemed greater after an unexpected than a to-be-attended prepulse. Indeed, analyzing the averaged event-related potentials (ERPs) ignores induced activity, i.e., event-related changes that do not appear or are poorly represented in the average (Delorme et al., 2002; Makeig et al., 2002). These authors have demonstrated that focusing analysis on average responses ignores: (i) event-related dynamics that do not appear in (or are poorly represented) response averages; and (ii) the ongoing EEG processes that may be partially time-and phase-locked by experimental events (and which thereby contribute portions of the average response). Hence, in the present study, attention seems to have specific effect on induced cortical oscillations but later. This observed desynchronization probably corresponds to a phenomenon other than sensory gating of the auditory elicited cortical oscillations and needs further investigation.

### Effects at the 1000 ms Prepulse-Pulse Interval

At a prepulse-pulse interval of 1000 ms, PPI of the spectral power was also observed. However, there was no specific influence of attention on the magnitude of PPI for the theta and beta2 frequency bands. This agrees with previous studies showing that at long-lead intervals, sensory gating is not modulated by controlled attentional processes. Indeed, Hazlett et al. (2001) and Rissling et al. (2007) reported that during a startle-CPT task with a prepulse-pulse interval of 1200 ms, there were no differences in eye-blink PPI when comparing a to-be-attended visual prepulse with a to-be-ignored visual prepulse. We have also previously observed the absence of a differential effect of attention at a long-lead interval for inhibition of the N100 and P200 components of the AEP (Annic et al., 2014). Nonetheless, our present results evidenced attentional modulation of PPI for the alpha band (with greater PPI after a to-be-attended prepulse and an unexpected prepulse than after a to-be-ignored prepulse) and for the beta1 band (with greater PPI after a to-be-attended prepulse than after a to-be-ignored prepulse). Thus, at a long-lead interval, goal-directed attention appears to modulate the inhibition of auditory-elicited alpha and beta1 oscillations, whereas stimulus-driven attention appears to modulate the inhibition of auditory-elicited alpha oscillations. This specific effect of attention on inhibition of cortical oscillations at long-lead intervals is rather surprising, since it was not observed for ERPs (Kedzior and Martin-Iverson, 2006; Kedzior et al., 2007; Annic et al., 2014).

### Effects at Attention on Phase Coherence

In terms of phase coherence, our results showed that the value of the ITC was the same at both prepulse-pulse intervals for the theta, beta1 and beta2 frequency bands—regardless of whether the pulse was presented alone (during the control task) or preceded by a prepulse (of whatever type). An effect of attention on ITC was only observed for the alpha band (regardless of the prepulse type). This partially counter our hypothesis in which the prepulse would impair phase synchronization. The lack of attentional modulation of ITC for the theta, beta1 and beta2 bands is not in agreement with the spectral power results—suggesting that inhibition of power is not due to an impairment in intertrial phase variability. This is in line with the fact that the ITC is independent of changes in EEG power (Makeig et al., 2004). Since the ITC is a measure of intertrial phase synchronization of EEG activity (Delorme and Makeig, 2004), the attenuated value for the alpha band during the startle-CPT task is probably related to general attentional demands during the task. In fact, it has been shown that desynchronization of the alpha band (7–10 Hz) is related to the levels of expectancy and attention (Pfurtscheller and Klimesch, 1992). Nevertheless, it is difficult to compare our results with the literature data, since our present study is the first to assess the effect of a prepulse on the phase synchronization of brain oscillations. Using a dual-click paradigm, Edwards et al. (2009) found that the ITC for the theta, alpha and beta band frequencies was lower for S2 than for S1. However, the experimental designs differed.

### Limitations

Our study had several limitations. Although our study population comprised both men and women, we lacked data on the latter’s hormonal status. In fact, Jovanovic et al. (2004) have shown that PPI varies according to the phase of the menstrual cycle. Moreover, Swerdlow et al. (1993) have demonstrated that PPI of the acoustic startle reflex was reduced in females compared to males. However, most recent studies of PPI in humans do not exclude females and have either failed to test for a sex effect (Rissling et al., 2005; Kedzior et al., 2006; Ashare et al., 2007; Molina et al., 2009; Scholes and Martin-Iverson, 2009; Larrauri et al., 2012) or have found that a sex effect was not present (Stojanov et al., 2003; Kedzior and Martin-Iverson, 2007; Kedzior et al., 2007; Rissling et al., 2007). Secondly, we did not confirm the self-reported absence of nicotine and cannabis use by assaying urine or blood samples. However, self-reporting of cannabis use is a validated way of evaluating actual consumption (Martin et al., 1988), and nicotine can even be detected in the urine samples of most of non-smokers (Matsukura et al., 1979; Baselt, 2000). Thirdly, we focused our PPI analysis on the time window corresponding to the “usual startle response” but our results showed a later event-related desynchronization mainly in the alpha band that seemed to be related to goal-directed attention and needs further investigation. Lastly, interpretation of our results is limited because the mechanisms involved in the regulation of the PPI of cortical oscillations are poorly known. Indeed, several animal studies have shown the role of the GABAergic, cholinergic and dopaminergic inhibitory neurons in the regulation of the PPI of the startle reflex (Koch et al., 1993; Fendt et al., 2001; Takahashi et al., 2007). However, these data only concern PPI of the motor response and even though we can speculate that similar mechanisms are involved in the regulation of the PPI of cortical responses, no data are available up to now. Further studies are thus warranted.

## Conclusion

Stimulus-driven attention and goal-directed attention both increase PPI of brain oscillations related to an irrelevant stimulus (the pulse). At a short-lead interval, stimulus-driven attention and goal-directed attention both modulate auditory-elicited theta, alpha and beta1 oscillations, whereas beta2 oscillations are preferentially inhibited when goal-directed attention is engaged. Our results also highlighted the impact of selective attention processes on sensory gating at long-lead intervals. Overall, it comes out of our results that auditory-elicited oscillations are globally modulated by attention whatever the frequency band, but that goal-directed attention has a specific effect on alpha and beta oscillations, with even a late desynchronization in the alpha frequency band. Our present findings do not suggest that phase synchronization of EEG activity is specifically modulated by attention. Given that PPI has been shown to be a useful index of sensory gating impairments in basal ganglia disorders [e.g., schizophrenia and Parkinson’s disease (Perriol et al., 2005; Hazlett et al., 2008b)], use of our active paradigm to study different frequency bands may enable a better understanding of the role of attention in these disorders.

## Author Contributions

Conception and design of the work, acquisition, analysis or interpretation for the work: AA, J-LB, CT, KD. Drafting the work or revising it critically for important intellectual content: AA, J-LB, AD, PB, CT, PD, KD. Final approval of the version to be published: AA, J-LB, AD, PB, CT, PD, KD. Agreement to be accountable for all aspects of the work in ensuring that questions related to the accuracy or integrity of any part of the work are appropriately investigated and resolved: AA, J-LB, AD, PB, CT, PD, KD.

## Conflict of Interest Statement

The authors declare that the research was conducted in the absence of any commercial or financial relationships that could be construed as a potential conflict of interest.

## Abbreviations

AEP: auditory evoked potential
CPT: continuous performance test
ERP: event-related potential
ERSP: event-related spectral perturbation
ITC: inter-trial coherence
PPI: prepulse inhibition.
